# A novel exomal ATRX mutation with preferential transmission to offspring: A case report and review of the literature for transmission ratio distortion in ATRX families

**DOI:** 10.3892/mmr.2020.11574

**Published:** 2020-10-09

**Authors:** Mariano Stabile, Davide Colavito, Elda Del Giudice, Anna F. Rispoli, Marina C. Ingenito, Anna K. Naumova

**Affiliations:** 1 Zygote Center, Center for Genetics, Prenatal Diagnosis, Fertility, I-84131 Salerno, Italy; 2 Research & Innovation s.r.l. (R&I Genetics), I-35127 Padova, Italy; 3 Departments of Obstetrics and Gynecology and Human Genetics, Research Institute of the McGill University Health Centre (RI-MUHC), McGill University, Montreal, Quebec H4A 3J1, Canada

**Keywords:** X-linked mental disability, *ATRX* mutation, SNF domain of ATRX, transmission ratio distortion

## Abstract

The present case report describes an Italian family with three affected probands, who exhibited serious mental disability, which has not been associated with other anomalies, except with slight facial dysmorphism. Molecular multigenic analysis for intellectual disability identified a previously unreported variant, p.Ile1765Met (c.5295C>G) in the SNF domain of the ATRX protein (in exon 24). The identified mutation was found in a hemizygous state in all three affected probands and in a heterozygous state in the asymptomatic mother and the female sibling. With respect to the phenotypic similarities found in the patients with those described in previous studies, the consistency in the mode of inheritance and segregation of the mutation, the variant reported in the present case report may be considered as ‘likely pathogenic’. To investigate the hypothesis that the preferential transmission of the *ATRX* mutation observed in this family reflected a general trend, a meta-analysis into the segregation of *ATRX* mutations from published pedigrees, following allelic transmission from mothers who are heterozygous carriers to their offspring, was performed. A preferential transmission of the mutant allele to male offspring (58% of males inherited the mutant allele) was found; however, the bias was not statistically significant (P=0.29; χ^2^ test).

## Introduction

X-linked mental disability (MRX) is a form of intellectual disability (ID) associated with the X chromosome. It is one of the most frequent forms of ID with an incidence rate of 3% in the human population.

One of the X-linked loci associated with MRX is the α thalassemia X-linked intellectual disability (*ATRX*) gene (OMIM #301040; Xq21.1); characterized by a complex phenotype and the primary elements consist of a serious impairment of psychomotor development, facial dysmorphism, genital anomalies and α thalassemia. Inherited mutations of *ATRX* are associated with an X-linked mental disability syndrome (XLMR), and the majority of cases also have α-thalassemia syndrome (ATR-X). ATRX syndrome, which has been associated with *ATRX* mutations is characterized by a complex phenotype, including serious impairment of psychomotor development, facial dysmorphism, genital anomalies and α thalassemia (1). Female carriers of *ATRX* mutations are intellectually normal and rarely show physical manifestations, due to an extreme skewing of X-inactivation in favor of the chromosome containing the non-mutant allele (1). There are >200 cases of ATR-X syndrome that have been identified using molecular diagnostics (2–5).

*ATRX* encodes a protein, consisting of 2,492 amino acids. It has multiple functions, including regulation of transcription, chromatin remodeling and ATP-dependent helicase activity (https://www.uniprot.org/uniprot/P46100). Alternative splicing of the gene results in distinct protein isoforms.

The protein has two primary functional domains, the N-terminal ADD domain and the C-terminal helicase/adenosine triphosphatase domain (ATPase), where the majority of reported mutations are located. Most of the reported mutations are missense, with c.536A>G and c.736C>T, both in the ADD domain, being the most common mutations found in different families. Mutations in the ADD domain are associated with greater impairment of psychomotor development, while most cases with mild/moderate mental disability have mutations in the helicase domain. The common mutation, c.736C>T, has been associated with low frequency of Hemoglobin H HbH (6).

In the present case report, a novel, likely pathogenic, *ATRX* variant c.5295C>G associated with mental disability has been identified. The family in the report shows a strong transmission bias in favor of the mutant allele: All 4 offspring inherited the mutant *ATRX* allele from their mother.

## Case report

Whole blood (3 ml) was collected for exome analysis after informed consent was provided. DNA was extracted using the Qiagen BioRobot DNA extraction kit (Qiagen Benelux B.V.) according to the manufacturer's instructions and quantified using NanoDrop spectral analysis (Thermo Fischer Scientific, Inc.). DNA integrity was evaluated using standard agarose gel electrophoresis (100 V; 30 min; 1.5% agarose gel in TBE buffer). DNA library preparation and whole exon enrichment were performed using Agilent All Exon V.6 kit (Agilent Technologies, Inc.). The library was sequenced using the HiSeq2500 Illumina Sequencer (125-bp paired end sequence mode; Illumina, Inc.). Bioinformatics analysis included the following: i) Next generation sequencing reads were mapped to whole genomes using the Burrows-Wheeler Alignment tool with the default parameters, ii) PCR duplicate removal using Picard (http://picard.sourceforge.net), iii) identification of single nucleotide polymorphisms and insertions/deletions using the Genome Analysis Toolkit (GATK) UnifiedGenotyper, iv) variant annotation using snpEff (http://snpeff.sourceforge.net) and v) false positive variant filtration using the GATK VariantFiltration module. Exome sequencing data and read alignment analysis was checked for coverage depth and alignment quality using the Bedtools software package. Variant classification was performed in accordance with the guidelines from the American College of Medical Genetics and Genomics (ACMG).

Phenotype driven analysis coupled with the employment of *in silico* multigene panels specific for ID and neurodevelopmental disorders was used to filter, select and interpret genetic variants obtained following exome sequencing. The presence of significant variants was confirmed using Sanger sequencing.

### 

#### Clinical description

**Figure 1.**
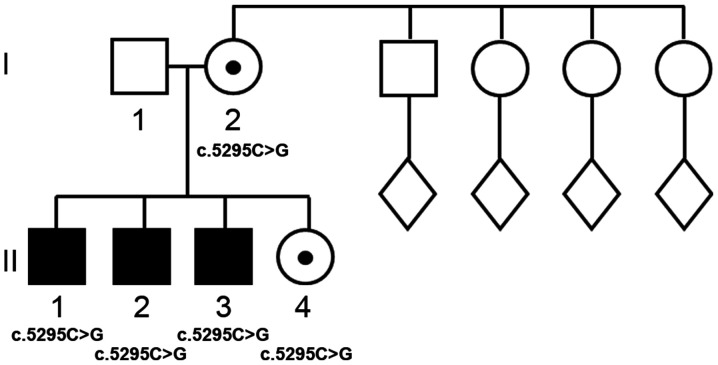
Family with 3 sons with mental disability. Family pedigree. Filled squares denote affected males. Filled dots denote carrier females. Open circles and squares denote unaffected individuals. The diamonds indicate the offspring of the germans of proband I-2.

In the Italian family A (Fig. 1) with healthy and non-consanguineous parents (I-1 and I-2), all three male probands (II-1, II-2, II-3) showed medium to severe mental disability and mild dysmorphism, while the heterozygous females (I-2 and II-4) are phenotypically and intellectually normal. The mother was 16, 18, 32 and 35-years-old when all of her children were born, respectively. The family history was unremarkable for ID or birth defects. In the mother's family, the male sibling was intellectually normal, as were the seven nephews born to the three female siblings (data not shown).

The three affected male probands have a similar clinical history and phenotype with greater intellectual impairment in one of the probands. They were delivered at term by caesarean section, following an uneventful pregnancy. Their birth weights were 3,200, 3,000, and 3,050 g for II-1, II-2 and II-3, respectively. The auxological parameters were within the limits of the normal range, while gross motor, cognitive and social milestones were delayed in early childhood. None of the probands had epileptic seizures. With respect to the characteristics associated with the pathology of ATRX syndrome, specific signs of facial dysmorphism (facial hypotonia and hypertelorism) were absent, while other signs (mouth ‘carp’ and depressed nasal bridge) were present. However, genital anomalies (cryptorchidism and/or ambiguous genitalia), epileptic seizures, autistic behavior, and α-thalassemia (microcytic anemia and Heinz bodies) were absent.

The first-born, II-1 (48 years old) lives at home with his family. Currently, the patient is autonomous in his movements. Partial presence of linguistic acquisitions and gestural communication is also present. The patient is very calm and has no problems with breathing or swallowing. There are no ‘snap’ movements of the upper limbs and enjoys care and physical contact. The other two probands have been hospitalized at a facility. The cranial magnetic resonance imaging performed on II-1 revealed cerebral hypoplasia without signs of cortical dysplasia, important dilatation of the subarachnoid spaces in the temporal and frontal regions, and large cisternal spaces in front of the trunk.

The second born, II-2 (46 years old) is the most intellectually and neurologically compromised. He does not control sphincters and physiological stimuli, and has no linguistic acquisition and gestural communication. Therefore, assessment of his intellectual level is not possible.

The youngest of the three male siblings, II-3 (32 years old) presents acquisition of language and gestural communication, as well as learning related to social and relational skills.

#### Laboratory analysis

The standard karyotypes of all the patients were normal. Fragile X screening, which was performed in the mother, all 3 male probands and the female sibling highlighted the presence of two alleles with values in the normal range. Genome analysis using the comparative genomic hybridization-array technique with SurePrintG3 ISCA V2 GCH60K microarrays (Cytogenomics v3.0.0.019; Agilent Technologies, Inc.) was performed on the mother and one of the probands (II-2). No imbalances in the number of copies of the analyzed genomic sequences, i.e. no deletions or duplications, were detected (data not shown).

Sequencing analysis of the coding exons of the genes included in the genetic panel for the molecular diagnosis of ID and neurodevelopmental disorders showed the presence of the hemizygous variant, c.5295C>G (resulting in the amino acid change p.Ile1765Met) in the *ATRX* gene in all three probands, while the unaffected mother and female sibling were heterozygous carriers of this variant.

This mutation was not present in allele frequency databases (ExAC, EVS and GnomeAD). Bioinformatics predictors indicate that isoleucine at position 1,765 is highly conserved through higher vertebrates (PhyloP-Primates, 2.70/6.42; PhyloP-Primate, 0.60/0.65) and the missense change, isoleucine to methionine is likely to be deleterious (Polyphen2, 1.00/1.00; SIFT, 0.001/0.00; CADD-Phred, 18). The analysis of the quaternary structure of the protein (pfam database) showed that the novel mutation, at position 1,765, falls within the SNF2 N helicase domain (Table I) located between the ADD and the helicase C domains. The SNF domain is found in proteins involved in a variety of different functions, including transcriptional regulation, recombination, and DNA repair (7).

Therefore, in the absence of further information, and in agreement with guidelines by ACMG (2015), the mutation should be considered of uncertain significance. We propose that, based on the overlap within the phenotypes of the probands, with those previously described, the concordance with the mode of inheritance, together with the result of the analysis of family segregation, the variant p.Ile1765Met (c.5295C>G) reported in the present study and found in a hemizygous state in all the affected subjects of the same family, may be considered ‘likely pathogenic’.

#### Meta-analysis of maternal transmission of ATRX mutations

Family A shows a striking bias in favor of transmission of the mutant allele from the mother who is a carrier to all four of her living children. Such bias may be due to i) a preferential transmission of mutant *ATRX* alleles; ii) the presence of a deleterious mutation on the X chromosome that carries the non-mutant *ATRX* allele in the mother; or iii) it could be purely stochastic.

To investigate the possibility that mutant *ATRX* alleles were preferentially transmitted to offspring, a meta-analysis of published pedigrees with complete reporting of family structures was conducted. Data on transmission of *ATRX* alleles for 42 mothers were extracted from papers published between 1991 and 2019 (8–21). Probands and index cases were excluded from the calculations, as well as offspring from one mosaic mother. A modest bias was found in favor of *ATRX* mutations being transmitted to male offspring. Among the total of 127 offspring, 54% inherited the mutation, while 58% of the 70 males inherited the mutant allele. However, the bias did not reach statistical significance (P=0.29; χ^2^ test). All data from families with ATRX were analyzed using the chi square test (χ^2^ test). A chi-square test is a statistical hypothesis test, used to determine whether there is a statistically significant difference between the expected frequencies and the observed frequencies in one or more categories of a contingency table. The chi-square test applied to our sample was P=0.29, which does not reach statistical significance.

Next, the analysis was limited to a subset of families where the mutations were identified, and all members of the family were genotyped (Table II) (8–12,14–17). A modest sex-ratio distortion was observed in favor of the male offspring, as well as transmission-ratio distortion in favor of the *ATRX* mutant alleles, but these did not reach statistical significance either (Table II).

It was proposed that the non-mutant X chromosome of the mother in family A was affected by a deleterious aberration limiting its transmission to offspring. However, no evidence of deletions or duplications, or X-autosomal translocations were found. Nevertheless, the possibility that she carries an unidentified deleterious mutation on the chromosome harboring the non-mutant *ATRX* allele cannot be ruled out.

## Discussion

The *ATRX* gene encodes a protein that belongs to the SNF2 chromatin remodeling protein family. Members of this large family of proteins modify the accessibility of DNA wrapped around a nucleosome, ensuring the nucleic acid is more or less accessible to proteins, which regulate the transcription process. To date, the exact role of ATRX is still under debate (22). ATRX contains two highly conserved domains, namely the ADD domain at the N-terminus and a C-terminal ATPase/helicase domain, the latter is involved in ATP hydrolysis. The ADD domain was named ARTX-DNMT3-DNMT3L, based on the sequence homology with a family of DNA methyltransferases. Argentaro *et al* (7) reported the solution structure of the ADD domain of ARTX, which consisted of an N-terminal GATA-like zinc finger, a plant homeodomain (PHD), and a long C-terminal α-helix. All the pathogenic mutations associated with X-linked mental disability map to the ADD and helicase domains.

In the present case report, a novel, likely pathogenic, missense mutation located in exon 24 of the major splice form of *ATRX* was identified. The novel p.lle1765Met variant changes the amino acid sequence of the SNF2 N helicase domain in the protein. This region is often affected by mutations associated with ATRX syndrome (23). Methionine is an α-amino acid and termed as a non-polar, aliphatic amino acid due to the S-methyl thioether side chain. Isoleucine is a non-polar, uncharged (at physiological pH) aliphatic amino acid with a branched chain. Isoleucine and methionine are both non-polar amino acids; however, due to their different structures, a modification of the tertiary structure and function of the SNF2 N domain cannot be excluded. The novel pIle1765Met mutation is also close to the highly conserved ATPase active site motif III. Another mutation, Leu1746Ser, which is located very close but outside of motif III, has been shown to uncouple the ATPase activity of ATRX from its ability to bind and translocate along DNA (23). In *in vitro* experiments, the mutant Leu1746Ser was more efficient at hydrolyzing ATP compared with that in the wild type variant, but failed to displace the third DNA strand in the triple helix displacement assay (23).

Considering the family tree of family A, a notable observation is that the *ATRX* mutation was transmitted to all four offspring from the carrier mother, while the composite probability of such a transmission is only 6.25%. We therefore hypothesized if the mutant *ATRX* allele was more likely to be transmitted to offspring by carrier mothers and conducted a meta-analysis of the segregation of *ATRX* mutations in published pedigrees (Table II). There was a tendency towards preferential transmission of the mutant allele to male offspring (58% of males inherited the mutant allele); however, the bias was not statistically significant (P=0.29, χ^2^ test). The transmission-ratio distortion and its underlying mechanisms are still poorly understood, with explanations ranging from meiotic drive (alleles preferentially retained in the oocyte vs. the polar body) to differences in embryo survival (24–26). It is worth noting that ATRX is essential for oogenesis and its loss affects normal chromosome segregation (27). However, even without full understanding of the underlying mechanisms, preferential transmission of mutant alleles may be an important aspect to be considered in genetic counseling.

## Figures and Tables

**Table I.** Table pfam domains.

Source	Domain	Start	End
Disorder	n/a	1	159
Low_complexity	n/a	24	33
Low_complexity	n/a	59	83
Low_complexity	n/a	103	114
Pfam	ADD ATRX	158	213
Disorder	n/a	311	312
Disorder	n/a	415	440
Disorder	n/a	445	581
Disorder	n/a	586	588
n/a	n/a	n/a	n/a
n/a	n/a	n/a	n/a
n/a	n/a	n/a	n/a
Pfam	SNF 2 N	1536	1889^a^
Disorder	n/a	1544	1545
Disorder	n/a	1551	1553
Low_complexity	n/a	1912	1925
Disorder	n/a	1913	1915
n/a	n/a	n/a	n/a
n/a	n/a	n/a	n/a
n/a	n/a	n/a	n/a
Pfam	Helicase C	2018	2155
Disorder	n/a	2218	2229
Disorder	n/a	2254	2266
Low_complexity	n/a	2261	2269
Low_complexity	n/a	2267	2282
n/a	n/a	n/a	n/a
n/a	n/a	n/a	n/a
n/a	n/a	n/a	n/a
Low_complexity	n/a	2466	2479

The table provides domain boundaries for the ATRX protein.

a Amino acid substitution in the SNF 2N domain in the family A. SNF, Sucrose Non-Fermentable. n/a, not applicable.

**Table II.** Meta-analysis of *ATRX* allele transmission from carrier mother to offspring in families with confirmed mutations.

				Sons		Daughters			
							
No.	Mutation	Pedigree	Mother	Carrier	Non-carrier	Carrier	Non-carrier	Proband/index Caseexcluded	Authors/(Refs.)
1	c.5295C>G			2	0	1	0	Yes	Current report
2	c.6130C>T			1	0	0	0	Yes	Altiner and Raymond 2019 (9)
3	c.515C>T		II-1	0	0	1	0		Li *et al* 2020 (14)
4	c.6257T >C	3	III-1	1	0	0	0		Yan *et al* 2019 (21).
5	c.6257T >C	3	III-4	1	0	0	1		Yan *et al* 2019 (21)
6	c.6718C>T			1	0	0	0	Yes	Thakur *et al* 2011 (17)
7	c.6740A>C			0	0	2	0	Yes	Bouazzi *et al* 2016 (11)
8	c.109C>T	K8035	I-2	1	4	2	0		Basehore *et al* 2015 (10)
9	c.109C>T	K8035	II-1	2	2	1	1		Basehore *et al* 2015 (10)
10	c.109C>T	K8035	III-1	1	2	1	1		Basehore *et al* 2015 (10)
11	c.109C>T	K8035	II-4	1	0	0	1	Yes	Basehore *et al* 2015 (10)
12	c.109C>T	K8360	I-2	2	2	2	1	Yes	Basehore *et al* 2015 (10)
13	c.109C>T	K8360	II-3	1	0	0	0		Basehore *et al* 2015 (10)
14	c.109C>T	K8820	I-2	4	4	1	1		Basehore *et al* 2015 (10)
15	c.109C>T	K8820	II-10	1	1	0	1	Yes	Basehore *et al* 2015 (10)
16	c.109C>T	K9574/MRX77	II-2	1	1	5	0		Basehore *et al* 2015 (10)
17	c.109C>T	K9574/MRX77	III-1	1	0	0	2		Basehore *et al* 2015 (10)
18	c.109C>T	K9574/MRX77	III-3	1	0	1	0		Basehore *et al* 2015 (10)
19	c.109C>T	K9574/MRX77	III-4	2	0	0	0		Basehore *et al* 2015 (10)
20	c.109C>T	K9574/MRX77	III-5	0	1	0	1		Basehore *et al* 2015 (10)
21	c.109C>T	K9574/MRX77	III-6	2	0	0	0		Basehore *et al* 2015 (10)
22	c.109C>T	JHH2445-6		1	0	0	0	Yes	Basehore *et al* 2015 (10)
23	c.109C>T		II	1	0	0	0	Yes	Moncini *et al* 2013 (16)
24	c.109C>T		IV-2	0	1	1	0	Yes	Abidi *et al* 2005 (8)
25	c.7156C>T			0	1	0	1	Yes	Masliah-Planchon *et al*
	Total			28	20	18	11		2018 (15)

Excess of male offspring (sex-ratio distortion) and mutation carriers (transmission-ratio distortion) is noted, but neither reach statistical significance: P=0.055 for sex-ratio distortion, P=0.087 for transmission-ratio distortion, χ^2^ test.

## Data Availability

The datasets used and/or analyzed during the current study are available from the corresponding author on reasonable request.
